# Potential Clinical Benefits of CBD-Rich *Cannabis* Extracts Over Purified CBD in Treatment-Resistant Epilepsy: Observational Data Meta-analysis

**DOI:** 10.3389/fneur.2018.00759

**Published:** 2018-09-12

**Authors:** Fabricio A. Pamplona, Lorenzo Rolim da Silva, Ana Carolina Coan

**Affiliations:** ^1^Entourage Phytolab, São Paulo, Brazil; ^2^Bedrocan Brasil, São Paulo, Brazil; ^3^UNICAMP, Campinas, Brazil

**Keywords:** cannabinoids, cannabidiol (CBD), epilepsy, meta-analysis, refractory epilepsy, phytotherapy

## Abstract

This meta-analysis paper describes the analysis of observational clinical studies on the treatment of refractory epilepsy with cannabidiol (CBD)-based products. Beyond attempting to establish the safety and efficacy of such products, we also investigated if there is enough evidence to assume any difference in efficacy between CBD-rich extracts compared to purified CBD products. The systematic search took place in February/2017 and updated in December/2017 using the keywords “epilepsy” or “Dravet” or “Lennox-Gastaut” or “CDKL5” combined with “Cannabis,” “cannabinoid,” “cannabidiol,” or “CBD” resulting in 199 papers. The qualitative assessment resulted in 11 valid references, with an average impact factor of 8.1 (ranging from 1.4 to 47.8). The categorical data of a total of 670 patients were analyzed by Fischer test. The average daily dose ranged between 1 and 50 mg/kg, with treatment length from 3 to 12 months (mean 6.2 months). Two thirds of patients reported improvement in the frequency of seizures (399/622, 64%). There were more reports of improvement from patients treated with CBD-rich extracts (318/447, 71%) than patients treated with purified CBD (81/175, 46%), with statistical significance (*p* < 0.0001). Nevertheless, when the standard clinical threshold of a “50% reduction or more in the frequency of seizures” was applied, only 39% of the individuals were considered “responders,” and there was no difference (*p* = 0.52) between treatments with CBD-rich extracts (122/330, 37%) and purified CBD (94/223, 42%). Patients treated with CBD-rich extracts reported lower average dose (6.0 mg/kg/day) than those using purified CBD (25.3 mg/kg/day). The reports of mild (158/216, 76% vs. 148/447, 33%, *p* < 0.001) and severe (41/155, 26% vs. 23/328, 7%, *p* < 0.0001) adverse effects were more frequent in products containing purified CBD than in CBD-rich extracts. CBD-rich extracts seem to present a better therapeutic profile than purified CBD, at least in this population of patients with refractory epilepsy. The roots of this difference is likely due to synergistic effects of CBD with other phytocompounds (aka Entourage effect), but this remains to be confirmed in controlled clinical studies.

## Introduction

Derivative products from the *Cannabis sativa* plant have historically been used for a number of neurological disorders, as broad as pain and appetite stimulation in oncological and HIV patients (1). The delicate balance between therapeutic and adverse effects in medicinal *Cannabis* yields controversial discussions in the literature (2–4), where the main psychoactive cannabinoid compound—delta-9-tetrahydrocannabinol (THC) is the major protagonist. More recently, another non-psychoactive cannabinoid–cannabidiol (CBD)—has received a lot of attention, since its promising pharmacological profile suggests a broader therapeutic index compared to THC. CBD has been described as a potential therapeutic compound to control seizures in humans in the 80's (5) and since then, several other studies have extended this data (6–15).

The natural source of CBD is a variety of *Cannabis* plants called “hemp” or “fiber-type *Cannabis*,” where one can find a high ratio between CBD and THC compounds, sometimes around 30:1 (CBD:THC), with negligible amounts of THC (16, 17). Fiber-type *Cannabis* are, by definition, *Cannabis* with < 0.3% THC content, which is not considered controlled substance by the United Nations Office on Drugs and Crime (18). Hemp extracts became an internet buzz (19), with several anecdotal descriptions of therapeutic effects in children with treatment-resistant epilepsies, especially Dravet syndrome, starting to appear since 2013 (11, 20, 21). Preclinical evidence support anti-convulsant properties of CBD [reviewed in Hill et al. (22) and Devinsky et al. (23)]. Furthermore, a number of observational papers suggested good tolerability and therapeutic benefits in seizure control, with patients experiencing low frequency of side effects (6–15). Few randomized control trials in specific diseases have followed (24, 25) and the putative neuronal mechanism of action is still to be established, with the more likely candidates being inhibition of endocannabinoid uptake, allosteric modulation of CB1 receptors, activation of 5-HT1A serotoninergic receptors anti-inflammatory/anti-oxidant effects [reviewed in Bih et al. (26)].

The first CBD-based product was just recently registered for the treatment of treatment-resistant epilepsies (27). Meanwhile, the patients are using non-registered hemp extracts and derivative products that are considered “nutritional supplements” with high CBD content and often unknown THC concentration. These products are not considered controlled substances at the production countries and are being distributed in many countries via exceptional import mechanisms.

Despite several positive anecdotal pieces of evidence of patients and family members about the “CBD extracts,” which are broadly publicized through several magazines and TV shows worldwide; until now, there is no consensus on the medical literature about the efficacy and safety of these products. Some observational studies are available on scientific literature, but there is a scarcity of clinical data acquired within the logic, rigor and organization necessary to the conduction of clinical studies destined to the registration of a pharmaceutical product.

The objective of the present study is to conduct a meta-analysis to investigate the available data about the clinical use of CBD-rich products for patients with treatment-resistant epilepsy. Whenever possible, we also tried to investigate if there was any difference of efficacy and side effects between “purified CBD” and CBD-rich extracts.

## Meta-Analysis Search Strategy

### Sources

A systematic search was performed on MEDLINE/PubMed (http://www.ncbi.nlm.nih.gov/pubmed) and Google Scholar (http://scholar.google.com) databases intending to identify original papers with clinical data (observational) on the use of *Cannabis* and its compounds on the treatment of refractory epilepsy. Main focus was on cannabidiol (CBD) and CBD-rich *Cannabis* extracts, whose use has been disseminated among infant and juvenile patients of treatment-resistant forms of epilepsy. The search was limited to papers published in English, with results obtained from human beings in parent surveys and proper medical records. The systematic search took place in February/2017 and updated in December/2017 using the keywords “child” and “epilepsy” or “Dravet” or “Lennox-Gastaut” or “CDKL5” combined with “Cannabis,” “cannabinoid,” “cannabidiol,” or “CBD.” We made every effort to include the available data, including searching through the paper references to identify additional sources, contacting the studies' authors and presenting a preliminary version of this study in two conferences to gather additional information that we could have missed in the first search. Papers containing only title and/or abstracts were not included, as well as unpublished results (at the time of the first search one in press article was included, but it is now officially published). Pre-print servers like PeerJ and BioRxiv were also used for the search, but did not reveal any forthcoming useful clinical study.

### Study Selection

The titles, abstracts and full texts of all search results had their eligibility analyzed, considering inclusion and exclusion criteria. Inclusion criteria: studies containing observational clinical data in humans that could infer the efficacy and/or safety profile of the products containing cannabinoids for epilepsy. Exclusion criteria: Review and opinion papers, case studies, studies with no measurable data, and studies with no accessible numerical data. Papers describing studies in prospective and retrospective design were considered eligible, regardless of the kind and duration of treatment. Papers failing to present objective measurement of seizures and/or objective measurements of clinical improvement were disregarded. Papers presenting partial data (example: data for clinical improvement, but not for adverse effects) were included only in the appropriate sections of the meta-analysis study.

### Treatment of Clinical Data and Statistical Analysis

Classical objective clinical outcomes in the research field of epilepsy were used to group the articles. Subjective clinical outcomes like “reported improvement” were considered, but the more objective “reduction of the number of seizures” was preferred. Data regarding the reduction in the seizures frequency were grouped in two cumulative thresholds, (1) reduction in seizures frequency >50% (classically considered a “responder” to the treatment in epilepsy studies) and (2) reduction in seizures frequency >70% were considered for objective measurement of treatment efficacy in the pooled data (whenever available). The relative number of “responders” in each study was used as the main objective measurement to evaluate efficacy and for comparison between treatment types (purified CBD vs. CBD-rich *Cannabis* extracts). Data were pooled together in categorical variable format (proportion of patients) for combined analysis. Data from papers using continuous variable format (percentage, or individual frequency reduction) were inferred/estimated and transformed in categorical variable for further analysis. In two cases, the authors were contacted for additional information that could not be inferred from the paper. The transformed data were analyzed statistically by the Fischer test for categorical variables. The data of every paper was organized in tables and plotted in RawGraphs (http://app.rawgraphs.io/) for the scatterplot diagram. Direct comparisons were performed among different epileptic encephalopathies (Lennox-Gastaut patients vs. whole epileptic population; Dravet syndrome patients vs. whole epileptic population) and between “purified CBD” and “CBD-rich extracts” preparations, whenever possible (Fischer test, *p* < 0.05).

As clinical safety outcomes, all reported outcomes of adverse events were considered and grouped afterwards by similarity. The objective data considered were the “frequency of adverse events,” categorized according to the symptoms and severity (“mild” or “severe”), according to the description in the original paper.

### Clinical Studies Considered in the Meta-Analysis

**Figure 1:**
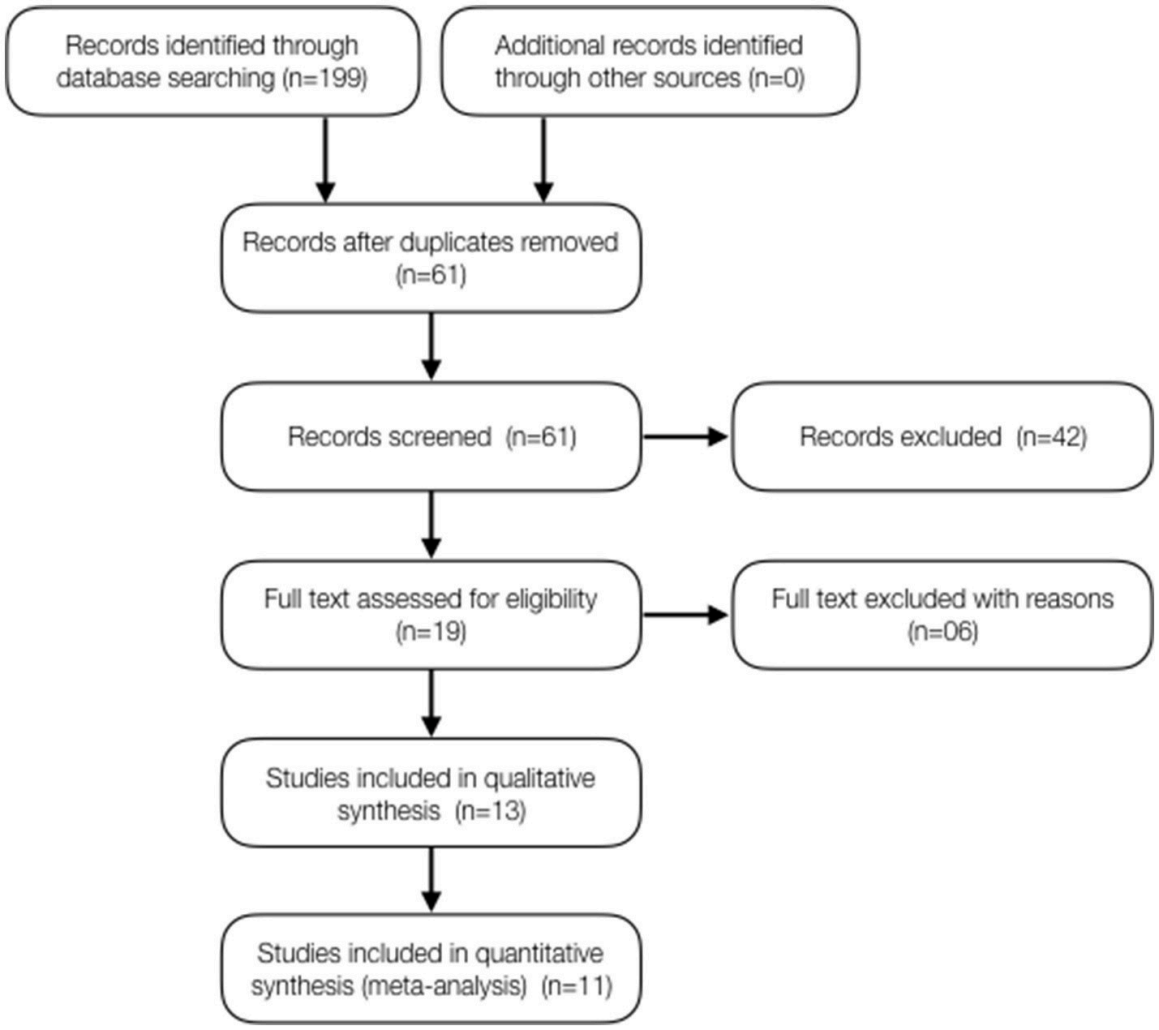
FLOW diagram of the reference searches.

The systematic search took place in February/2017 and updated on the 13th of December 2017 using the keywords “child” and “epilepsy” or “Dravet” or “Lennox-Gastaut” or “CDKL5” combined with “Cannabis,” “cannabinoid,” “cannabidiol,” or “CBD” resulting in 199 papers. From these, 138 were duplicates and were removed. The remained 61 records were screened and 42 of these studies were excluded. Nineteen (19) papers were assessed for eligibility and 6 papers were excluded due to lack of observational clinical data (ex: preclinical studies). The qualitative assessment of 13 articles resulted in 11 valid references for analysis, with an average impact factor of 8.1 (ranging from 1.4 to 47.8) (Figure 1).

All the studies included are fairly recent, published between 2013 and 2017, showing how vivid this subject is in the scientific literature worldwide. Overall, the papers analyzed report observational clinical data from 670 patients, treated with CBD-rich *Cannabis* extracts or purified CBD, with average daily doses between 1 and 50 mg/kg, and duration of treatment from 3 to 12 months (average of 6.5 months), as shown in Table 1 below.

**Table 1:** Information about the clinical studies included as valid reference in the current meta-analysis.

**Treatment references**	**Medical center**	**Study design**	**Patients**	**Age (year)**	**Duration**
CBD pure (6)	Langone Med. Center NY Univ. (USA)	Prospective medical record	137	10.5 year (1–30)	3 months
CBD pure (7)	Child Neurology, Child Hosp. Philadelphia (USA)	Prospective medical record	7	NR	12 months
CBD pure (8)	Pediatric Epilepsy, Mass. Gen. Hospital-Harvard (USA)	Prospective medical record	13	10.8 year (4–19)	2 months
CBD pure (9)	Pediatric Epilepsy, Mass. Gen. Hospital-Harvard (USA)	Prospective medical record	18	14 year (2–31)	~9 months (6–12+)
CBD pure (10)	Langone Med. Center NY Univ. (USA)	Prospective medical record	48	11.7 year (1–30)	3 months
CBD-rich extract (11)	Neurology, Stanford (USA)	Parent survey (online)	19	6.2 year (2–16)	8 months
CBD-rich extract (12)	Pediatric Neurology, Univ. Calif. Los Angeles-UCLA (USA)	Parent survey (online)	117	6 year (0.4–NR)	6.8 months
CBD-rich extract (28)	Pediatrics and Neurology, Univ. Colorado (USA)	Retrospective medical record	75	7.3 year (0.5–18)	5.6 months
CBD-rich extract (13)	Pediatric Neurology, Sheba Medical Center (Israel)	Retrospective medical record	74	~10 year (1–18)	6 months
CBD-rich extract (14)	Inst. Tec. Est. Sup. Monterrey (Mexico)	Parent survey (online)	53	~9.4 year (0.8–18)	4.2 months (1–12)
CBD-rich extract (15)	Pediatrics and Neurology, Univ. Colorado (USA)	Retrospective medical record	119	7.5 year (0.1–18)	11.7 months

*Average treatment duration*.

From the selected studies, six (6) show a retrospective design (with a total of 447 patients) and 5 show a prospective design (with a total of 223 patients). The quality of evidence reported in the papers is a relevant variable: three (3) studies used research based on online questionnaires with family members and caretakers (179 patients) and 8 studies report evidence from proper medical history (491 patients). As for the type of treatment, five (5) studies report data from patients who used purified CBD (223 patients) and 6 studies report data from patients who used *Cannabis* extracts with high CBD content, whose composition is not standardized (466 patients). Noteworthy, these variables don't seem to constitute an obvious bias compromising interpretation of data, since the groups are relatively well balanced. The only remarkable difference is that all studies using purified CBD had a prospective design, while the studies using CBD-rich extracts had a retrospective design. All studies were conducted by medical centers experienced in conducting this type of study, at universities or internationally reputed research centers. Curiously, nine (9) out of the 11 studies were conducted or lead by universities or research centers in the United States (553 patients). One study was conducted in Israel (74 patients) and another in Mexico (43 patients). All studies used a heterogeneous population of epilepsy patients, and the segmentation in specific types of syndromes was eventually done afterwards (Figure 1).

The majority of the studied population consisted of children and adolescents, between 1 (one) and 18 (eighteen) years old with treatment-resistant epilepsy (refractory epilepsy), who tried between 4 (four) and 12 (twelve) other medications for 3 (three) years before trying CBD-based treatments. Needless to say, this population constitutes a very hard-to-treat population, diagnosed with treatment-resistant epileptic syndromes. Roughly, this affects one-third of the total population of epileptic patients.

## Results

**Figure 2:**
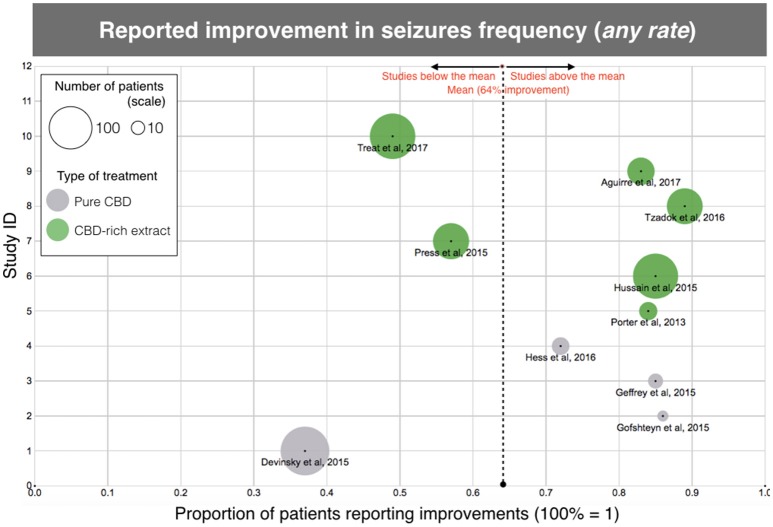
Scatterplot diagram of treatment efficacy, according to the data of “clinical improvement” reported in the aforementioned clinical studies. The X-axis represents the rate of clinical improvement (from 0 to 1, 100% = 1). The Y-axis is arbitrary “Study ID.” The size of each point represents the number of patients included in the study and gives an idea of the “weight” of each study. The dotted line is the average, regardless of treatment. Each type of treatment (Purified CBD vs. CBD-rich extracts) is coded with a different color, according to the legend. Same data as Table 1, except for one study (10) due to unreported data.

The results of efficacy in the studied population suggest that treatment with CBD-based products significantly reduces seizure frequency, even for this otherwise treatment-resistant population. According to the analysis of “reported improvement,” which means, any improvement reported in the selected papers, almost 2/3 of the patients had an observed reduction in seizure frequency (399/622, 64%), with individual studies rate ranging between 37 and 89% (Table 2; Figure 2). Notably, 6 out of 11 studies showed over 80% of the patients reporting improvement. There was a higher number of patients reporting improvement after using CBD-rich *Cannabis* extracts (318/447, 71%) than those treated with purified CBD (81/175, 46%), with valid statistical significance (*p* < 0.0001).

**Table 2:** Efficacy of treatments in the reduction of convulsive seizures (heterogeneous population).

**References**	**Patients**	**Reported improvement**	**>50%**	**>70%**	**Mean daily dose (mg/kg/day)**
**Total reports**	**670**	**399/622**	**216/553**	**83/311**	**(2–50 mg/kg)**
**Mean**	**100%**	**64%**	**39%**	**27%**	**15.0 mg/kg**
CBD pure (6)	137	37%	37%	22%	22.9 mg/kg
CBD pure (7)	7	86%	71%	57%	22 mg/kg
CBD pure (8)	13	85%	70%	46%	24.6 mg/kg
CBD pure (9)	18	72%	50%	22%	37.7 mg/kg
CBD pure (10)	48	NR	42%	NR	28.2 mg/kg
CBD-rich extract (11)	19	84%	74%	42%	7.0 mg/kg
CBD-rich extract (12)	117	85%	NR	NR	4.3 mg/kg
CBD-rich extract (28)	75	57%	33%	NR	NR
CBD-rich extract (13)	74	89%	34%	18%	<10 mg/kg
CBD-rich extract (14)	43	83%	67%	42%	3.2 mg/kg
CBD-rich extract (15)	119	49%	24%	NR	NR

*Endpoints: any improvement reported, improvement > 50% (“clinical responder”) and >70%, and average dose reported. NR, not reported; ?, inconclusive*.

However, when the clinical threshold of “reduction of 50% or more in seizure frequency” was evaluated, only 39% of the individuals were considered responders (studies varying between 24 and 74%), and there was no difference (*p* = 0.52) between treatments with CBD-rich extracts (122/330, 37%) and purified CBD (94/223, 42%). The mean dose, regardless of treatment was 15.0 mg/kg/day of CBD equivalent. The average daily dose reported for purified CBD was 25.3 mg/kg/day, while the average daily dose of CBD equivalent reported for CBD-rich *Cannabis* extract was merely 6.0 mg/kg/day.

Moreover, there was no difference among subtypes of epileptic encephalopathies (Dravet and Lennox-Gastaut syndromes), although the data implies that patients from these two genetic-related epileptic syndrome are more sensitive to CBD treatment (Supplementary Table 1). At least 27% of all treated patients showed an “improvement >70%” in seizures frequency (83/311 patients) and the studies varied between 18 and 57% (Table 2). The “seizure free” endpoint was not used for the analysis because it is not a parameter used by a significant amount of the selected studies. The number of individuals that remained free of seizures was close to 10% in the papers reporting this endpoint, which is a relevant amount for a population that tried several prior anti-epileptic medications without success. The proportion of patients reporting any improvement and “classical” responders was also relatively high for a treatment-resistant population and is slightly above the number of responders commonly observed in studies of registered anti-epileptic drugs (29–31).

Beyond the direct therapeutic effect of CBD in reducing epileptic seizures, reports about improvement in “secondary” health aspects were very common. They shall not be negligible, since they provide a significant improvement in quality of life for the patients and their family members. Secondary endpoints were reported for 285 patients in the selected papers. Unfortunately, not all studies considered such endpoints during their development. The main secondary effects were improvements in awareness (147/285, 52%), quality of sleep (88/285, 31%), mood (87/285, 30%), behavior/aggression (56/285, 20%), language/cognition (19/285, 7%), and motor skills (19/285, 7%) (Table 3). There were also reports of other improvements, but for the sake of the current meta-analysis, only those affecting at least 5% of the studied population were considered. Arguably, these effects occurred as a consequence of the seizures reduction, but in many cases they occurred before or even in the absence of significant reductions of epileptic seizures (considering each case individually). There were no reports of secondary health aspects in studies of purified CBD (6–10). However, it's impossible to conclude that no improvements on secondary endpoints occurred with this type of treatment; rather, it is more likely that the study didn't focus on this clinical phenomenon. As demonstrated in one of the studies with 117 patients (12), in a direct comparison of the same population, the conventional antiepileptic drugs caused an improvement in these “secondary” parameters related to quality of life, but this effect occurred in a smaller scale than with CBD-based treatments. This is true, at least, for the effects in mood improvement, awareness, sleep quality, and self-control. This data suggests that secondary positive events described for CBD are attributed to this substance, and not only due to the reduction in the frequency of seizures (Table 3).

**Table 3:** Positive secondary effects^*^ of treatment with CBD-rich *Cannabis* extracts and purified CBD described as secondary endpoints in the clinical studies.

**Treatment references**	**Patients**	**Mood**	**Alertness**	**Behav**.	**Sleep**	**Language**	**Motor**
**Total reports**	**508**	**151/226**	**275/508**	**91/224**	**150/447**	**51/149**	**28/268**
**Mean**	**100%**	**67%**	**54%**	**41%**	**34%**	**34%**	**10%**
CBD pure (9)	9/14	NR	86%	67%	NR	NR	NR
CBD pure (10)	47	57%	68%	42%	NR	67%	NR
CBD-rich extract (11)	19	79%	74%	32%	68%	NR	NR
CBD-rich extract (12)	117	63%	71%	NR	53%	NR	NR
CBD-rich extract (28)	75	NR	33%	33%	7%	11%	11%
CBD-rich extract (13)	74	NR	34%	34%	11%	15%	15%
CBD-rich extract (14)	43	83%	89%	NR	77%	NR	NR
CBD-rich extract (15)	119	NR	39%	NR	7%	NR	8%

* *Only those reported by at least 5% of the study population are listed. NR, not reported*.

Although treatment with CBD products is regarded as at least equally safe in comparison to regular anti-epileptic drugs, CBD is not devoid of adverse effects (11). The studies mention the occurrence of adverse events on a relatively large portion of the population studied (217/422, 51%), even though the great majority of events are considered “mild.” Severe events were reported by a smaller portion of patients (64/422, 15%) (11). Importantly, in this case, we are considering only patients of studies that mentioned the occurrence of adverse effects. To improve accuracy, if the study did not mention adverse events, we considered that it was not reasonable to assume that there were no adverse events and, therefore, the whole study was excluded of the analysis. Two studies containing only 20 patients were excluded according to this criterion (7, 8). Counter-intuitively, there is also an advantage of CBD-rich extracts in relation to purified CBD regarding the occurrence of adverse events. The reports of mild (158/216, 76% vs. 148/447, 33%, *p* < 0.001) and severe (41/155, 26% vs. 23/328, 7%, *p* < 0.0001) adverse effects were more frequent in products containing purified CBD that in CBD-rich extracts. The most common adverse events reported were appetite alteration, sleepiness, gastrointestinal disturbances/diarrhea, weight changes, fatigue, and nausea. Uncommon or rare adverse events include thrombocytopenia, respiratory infections and alteration of the liver enzymes (Table 4).

**Table 4:** Negative secondary effects of treatment with CBD-rich *Cannabis* extracts and purified CBD described as secondary endpoints in the clinical studies.

**References**	***n***	**Mild AE**	**Serious AE**	**Total AE**
**Total reports**	**663**	**308/663**	**64/483**	**326/663**
**Mean**	**100%**	**46%**	**13%**	**49%**
CBD pure (6)	137	79%	30%	128/137
CBD pure (9)	18	67%	0%	12/18
CBD pure (8)	13	77%	NR	10/13
CBD pure (10)	48	58%	NR	28/48
CBD-rich extract (11)	19	37%	0%	7/19
CBD-rich extract (12)	117	30%	0%	35/117
CBD-rich extract (28)	75	44%	13%	33/75
CBD-rich extract (13)	74	46%	18%	34/74
CBD-rich extract (14)	43	37%	0%	16/43
CBD-rich extract (15)	119	19%	NR	23/119

^*^Reporting adverse events in a study population does not necessarily mean that it is related to treatment. NR, not reported.

## Discussion

The present meta-analysis study confirms the anecdotal evidence that CBD treatment improves seizure control in patients with treatment-resistant epilepsy. Pooled together, the data from 11 studies provide strong evidence in support of the therapeutic value of high-CBD treatments, at least as far as this population of 670 patients is regarded. Important to say, not every study reported all the clinical parameters (e.g., % of responders, side effects, quality of life endpoints, etc.), therefore, the analysis might be skewed in some way that it's impossible to account for. The difference in the quality of the studies is also an important limitation that should be taken into consideration.

This said, it's clear that CBD works for this type of epilepsy, with over 60% of volunteers describing clinical improvement and nearly 40% being clinical responders at a hard threshold of over 50% reduction in seizure frequency (Table 2, this study). With the observational non-blinded design, it's impossible to quantify how much of this response would be due to placebo effect. It's common to see placebo effects ranging from 15 to 25% in well-conducted epilepsy studies (24, 25), but a recent clinical study surprisingly showed a placebo effect as high as 40% (32). This might suggest a big impact of the belief in the current “fashionable” therapy using cannabinoids on reported therapeutic responses. More objective physiological measures would help to improve accuracy and are welcome in cannabinoid-related clinical studies.

One remarkable observation of this study is the difference in average dose reported by patients taking “plant-based” and “purified” CBD treatments. Curiously, even though treatment with CBD-rich extracts and purified CBD yielded similar, the patients treated with CBD-rich extracts reported a significantly lower average daily dose than patients using purified CBD. As described in the results section, the average dose described by patients taking CBD-rich *Cannabis* extracts was over 4 times lower than the dose reported by patients taking purified CBD (Table 2). This data suggests that CBD is 4 times “more potent” when administered in herbal form, probably because other minor compounds present in the extract may contribute to its therapeutic effect (33). The interpretation of higher potency of CBD in combination with other minor compounds is in line with previous reports of synergistic effects between cannabinoid and even non-cannabinoid compounds (34).

For instance, King et al. (35) described a clear synergistic effect of the combination between CBD and THC, where THC potentiates CBD effects in a mouse model of neuropathic pain in substantially smaller dose range than when CBD is given alone. This was already described in the classic study by Karniol and Carlini where CBD blocked certain effects of THC: catatonia in mice, corneal arreflexia in rabbits, increased defecation and decreased ambulation in rats in the open field after chronic administration, and aggressiveness in rats after REM-sleep deprivation. In contrast, CBD potentiated THC analgesia in mice and the impairment of rope climbing in rats (36). Similar examples of pharmacological interaction between these cannabinoids were summarized in Russo and Guy (37). Another interesting aspect of cannabinoid pharmacology is that CBD tends to block some of adverse events of THC, like anxiety and paranoia (38). Modern pharmacology suggests that these effects are due to allosteric modulation of CB1 cannabinoid receptors by CBD (39, 40). This means that CBD exerts a “fine-tuning” of the CB1 cannabinoid receptor affecting the interaction of other cannabinoids at the receptor level. Noteworthy, the original description of the physiology of allosteric modulation of these receptors was performed by the main author of the current paper (41). Whether or not this mechanism contributes to the anti-epileptic effects of cannabinoid remain to be established, but preliminary evidence of CBD/THC synergistic interaction in a mouse model of refractory epilepsy were recently reported in the congress of the International Cannabinoid Research Society [Anderson et al. (42) oral presentation in ICRS]. On top of this, minor plant cannabinoids like canabidivivarin (CBDV), tetrahydrocannabivivarin (THCV), and cannabinol (CBN) are also anti-convulsants (43–48).

When it comes to adverse events, the same pattern was true: patients treated with CBD-rich extracts tend to show less adverse events, regardless of its severity. This is counter-intuitive, and we believe that it might be secondary to the dose. Since patients taking CBD-rich extracts reported lower CBD dose, it's reasonable to expect lower side effects, including those related with the oil vehicle itself, like gastrointestinal discomfort or abdominal pain.

The most common adverse events reported were appetite alteration, sleepiness, gastrointestinal disturbances/diarrhea, weight changes, fatigue, and nausea. Uncommon or rare adverse events include thrombocytopenia, respiratory infections, and alteration of the liver enzymes. There was a worsening of the seizure burden in some cases, but this is uncommon and cannot be necessarily attributed to the treatment. Uncommon or rare events reported occurred in combination with other anti-epileptic medication, particularly valproic acid and clobazam, and may be related to drug interaction, and not due to direct CBD toxicity. Data from a recent study suggested that CBD tends to reduce the occurrence of adverse events, in general, when used as an add-on therapy to other anti-convulsants (12). In that cohort with 117 patients, CBD reduced the occurrence of fatigue, sleepiness, irritability, insomnia, appetite loss, aggressiveness, nausea, dizziness, anxiety, confusion, weight loss, vomiting, and obsessive behavior in 5–10 times. Among an extensive list, the only adverse events that actually increased with the addition of CBD were weight gain and increased appetite (about 2 times higher).

In conclusion, this meta-analysis suggests that treatments using CBD are effective and safe, at least in the population of patients with treatment-resistant epilepsy, considering risks, and benefits inherent to the treatment of this severe neurological condition. A considerable share of patients obtains benefits from this treatment, and the adverse events, when they occur, are fairly mild. Apparently, CBD-rich *Cannabis* extracts are more potent and have a better safety profile (but not higher efficacy) than products with purified CBD. The lack of standardization among *Cannabis* extracts does not allow us to infer which characteristics of the product provide this therapeutic advantage. However, considering the scientific literature describing the “entourage effect” in plant compounds (34–37) and in the endocannabinoid system (41, 49), it's reasonable to suggest that the higher potency of the CBD-rich *Cannabis* extracts over purified CBD is related to other plant compounds acting synergistically to CBD, as discussed above. Controlled studies with standardized *Cannabis* extracts are necessary to confirm if these compounds contribute *per se* or synergistically for the anticonvulsive effect of *Cannabis* and its derivatives.

## Author Contributions

FP gathered data, analyzed results, and wrote the manuscript. LdS and AC helped with data organization and manuscript writing.

### Conflict of Interest Statement

FP is responsible for the development of Cannabis-based products at Entourage Phytolab. AC received monetary compensation for consulting work performed for Entourage Phytolab. LdS works at Bedrocan.
