# Corrigendum: Potential Clinical Benefits of CBD-Rich *Cannabis* Extracts Over Purified CBD in Treatment-Resistant Epilepsy: Observational Data Meta-analysis

**DOI:** 10.3389/fneur.2018.01050

**Published:** 2019-01-10

**Authors:** Fabricio A. Pamplona, Lorenzo Rolim da Silva, Ana Carolina Coan

**Affiliations:** ^1^Entourage Phytolab, São Paulo, Brazil; ^2^Bedrocan Brasil, São Paulo, Brazil; ^3^UNICAMP, Campinas, Brazil

**Keywords:** cannabinoids, cannabidiol (CBD), epilepsy, meta-analysis, refractory epilepsy, phytotherapy

In the original article, there was a mistake in Table 2 as published. The total number of one reference was wrongly included in the calculation of the endpoint efficacy over 70%. Where it reads 83/430, it should be 83/311, therefore 27% (instead of 19%). The corrected Table 2 appears below.

**Table 2:** Efficacy of treatments in the reduction of convulsive seizures (heterogeneous population).

**References**	**Patients**	**Reported improvement**	**>50%**	**>70%**	**Mean daily dose (mg/kg/day)**
**Total reports**	**670**	**399/622**	**216/553**	**83/311**	**(2–50 mg/kg)**
**Mean**	**100%**	**64%**	**39%**	**27%**	**15.0 mg/kg**
CBD pure (6)	137	37%	37%	22%	22.9 mg/kg
CBD pure (7)	7	86%	71%	57%	22 mg/kg
CBD pure (8)	13	85%	70%	46%	24.6 mg/kg
CBD pure (9)	18	72%	50%	22%	37.7 mg/kg
CBD pure (10)	48	NR	42%	NR	28.2 mg/kg
CBD-rich extract (11)	19	84%	74%	42%	7.0 mg/kg
CBD-rich extract (12)	117	85%	NR	NR	4.3 mg/kg
CBD-rich extract (28)	75	57%	33%	NR	NR
CBD-rich extract (13)	74	89%	34%	18%	<10 mg/kg
CBD-rich extract (14)	43	83%	67%	42%	3.2 mg/kg
CBD-rich extract (15)	119	49%	24%	NR	NR

*Endpoints: any improvement reported, improvement > 50% (“clinical responder”) and >70%, and average dose reported. NR, not reported; ?, inconclusive*.

In the original article, there was a mistake in Table 4 as published. Data from one reference was missing in the calculation of the endpoints mild AE and severe AE. Where it reads 285/663, it should be 308/663, therefore 46% (instead of 43%). Where it reads 64/487, it should be 64/483. The corrected Table 4 appears below.

**Table 4:** Negative secondary effects of treatment with CBD-rich *Cannabis* extracts and purified CBD described as secondary endpoints in the clinical studies.

**References**	***n***	**Mild AE**	**Serious AE**	**Total AE**
**Total reports**	**663**	**308/663**	**64/483**	**326/663**
**Mean**	**100%**	**46%**	**13%**	**49%**
CBD pure (6)	137	79%	30%	128/137
CBD pure (9)	18	67%	0%	12/18
CBD pure (8)	13	77%	NR	10/13
CBD pure (10)	48	58%	NR	28/48
CBD-rich extract (11)	19	37%	0%	7/19
CBD-rich extract (12)	117	30%	0%	35/117
CBD-rich extract (28)	75	44%	13%	33/75
CBD-rich extract (13)	74	46%	18%	34/74
CBD-rich extract (14)	43	37%	0%	16/43
CBD-rich extract (15)	119	19%	NR	23/119

^*^Reporting adverse events in a study population does not necessarily mean that it is related to treatment. NR, not reported.

In the original article, due to the errors in Tables 2, 4 mentioned above, corrections have been made to the **Abstract** as well as the **Results**, paragraphs one, two, three and five:

- “81/223, 36%” changed to “81/175, 46%” in Abstract and Results, paragraph one.

- “*p* = 0.56” changed to “*p* = 0.52” in Abstract and Results, paragraph two.

- “97/255, 38%” changed to “122/330, 37%” in Abstract and Results, paragraph two.

- “6.1 mg/kg/day” changed to “6.0 mg/kg/day” in Abstract and Results, paragraph two.

- “27.1 mg/kg/day” changed to “25.3 mg/kg/day” in Abstract and Results, paragraph two.

- “(109/285 vs. 291/346, *p* < 0.0001) and severe (23/285 vs. 77/346, *p* < 0.0001)” changed to “(158/216, 76% vs. 148/447, 33%, *p* < 0.001) and severe (41/155, 26% vs. 23/328, 7%, *p* < 0.0001)” in Abstract and Results, paragraph five.

- “17.7 mg/kg/day” changed to “15.0 mg/kg/day” in Results, paragraph two.

- “18%” changed to “27%” in Results, paragraph three.

- “83/430” changed to “83/311” in Results, paragraph three.

- “(Table 4)” changed to reference “(11)” in Results, paragraph five.

The aut hors apologize for this error and state that this does not change the scientific conclusions of the article in any way. The original article has been updated.

