# Supplementary material for: Potential Clinical Benefits of CBD-Rich Cannabis Extracts Over Purified CBD in Treatment-Resistant Epilepsy: Observational Data Meta-analysis
Source: Front Neurol. 2018 Sep 12;9:759. doi: 10.3389/fneur.2018.00759 (PMC6143706; doi:10.3389/fneur.2018.00759)
Supplement: Supplementary file 1 [file Table_5.docx]

**Tables**

**Table Efficacy per Disease, focus on Dravet and Lennox-Gastaut**

Supplementary Table 1 – Efficacy reported as the number of clinical responders (improvement above 50%) among genetic encefalopathies

| **Treatment**  *Reference* | **Epileptic syndrome** | **Responders** (> 50% improvement) | **%** |
| --- | --- | --- | --- |
| *Devinsky + Tzadok + Press + Porter +* | **Refractory epilepsy**  (combined studies) | **216/553** | **39%** |
|  | **Dravet syndrome** |  |  |
| CBD-rich extract *Porter et al, 2013* | Dravet syndrome | 7/12 | 58% |
| CBD pure *Devinsky et al, 2015* | Dravet syndrome | 16/32 | 50% |
| CBD-rich extract *Hussain et al, 2015* | Dravet syndrome | 11/15 | 73% |
| CBD-rich extract *Press et al, 2015* | Dravet syndrome | 3/13 | 23% |
| CBD-rich extract *Treat et al, 2017* | Dravet syndrome | 1/17 | 06% |
|  | **Responders** | **38/89** | **48%** |
|  | **Lennox-Gastaut syndrome** |  |  |
| CBD pure *Devinsky et al, 2015* | Lennox-Gastaut syndrome | 11/30 | 37% |
| CBD-rich extract *Press et al, 2015* | Lennox-Gastaut syndrome | 8/9 | 89% |
| CBD-rich extract *Treat et al, 2017* | Lennox-Gastaut syndrome | 11/19 | 58% |
|  | **Responders** | **89/207** | **43%** |
